# Prevalence of perianal diseases and associated factors in primigravida women: A hospital-based cross-sectional study

**DOI:** 10.15537/smj.2022.43.10.20220198

**Published:** 2022-10

**Authors:** Hasan Cantay, Ulku Ayse Turker

**Affiliations:** *From the Department of General Surgery (Cantay), Faculty of Medicine, Kafkas University, and from the Obstetrics and Gynecology Clinic (Turker), Kars Harakani Public Hospital, Kars, Turkey.*

**Keywords:** anorectal disease, constipation, hemorrhoid, pregnancy, primigravida

## Abstract

**Objectives:**

To define the incidence of anorectal diseases in primigravida women as well as in the first month after delivery and the factors affecting the development of anorectal diseases.

**Methods:**

The sample size was determined as 328 primigravida women. Research data were collected from pregnant women who applied to Gynecology and Obstetrics Polyclinic, Kars Harakani State Hospital Gynecology and General Surgery Polyclinic, Kafkas University, Kars, Turkey, between November 2020 and August 2021.The pregnant women were examined and surveyed 4 times; in the second and third trimesters, on the day after delivery, and in the first month after delivery. Chi-square test was used in the analysis of the data determined by counting. Variables which were statistically significant (*p*<0.05) in the Chi-square test were included in the backward logistic regression analysis.

**Results:**

Perianal disease was observed in 103 (38.6%) of the pregnant women. The incidence of perianal disease was found to be 4.917 times (confidence interval [CI]: [2.134-11.327]) higher in those with perianal disease compared with those without, 2.936 times (CI:[1.584-5.439]) higher in those who did not consume fiber-rich foods compared with those who did, 9.512 times (CI: [4.583-19.742]) higher in those with constipation compared with those without, and 23.721 times (CI: [5.363-104.915]) higher in those whose pushing stage duration was above average compared with that in those whose pushing stage duration was below average.

**Conclusion:**

In primigravida pregnants, the risk of perianal disease increases in those who have perianal disease before pregnancy, those who do not consume fibrous food, those who have constipation, and those who have a long pushing period.

**A**nal fissure, prolapsed hemorrhoids, external hemorrhoid thrombosis, and prolapsed internal hemorrhoid thrombosis are defined as anorectal diseases. According to literature, the incidence of anorectal diseases in the general population is 5-15%.^1,2^ The incidence of anorectal diseases during pregnancy may increase up to 44%. The most common anorectal diseases are hemorrhoids and anal fissures.^3-5^

A combination of several factors makes anorectal diseases a common occurrence in pregnancy. Intra-abdominal pressure increases in pregnant women, the enlarged uterus prevents venous return, muscles of the veins become weak because of the increased progesterone levels in pregnancy, and the gastrointestinal system activity slows down. All these factors lead to an increase in anorectal diseases during pregnancy.^2,4-7^

There is limited data in literature on the development of anorectal diseases based on gestational weeks in pregnant women. The present study aims to define the incidence of anorectal diseases in primigravida women as well as in the first month after delivery and the factors affecting the development of anorectal diseases.

## Methods

This study is a hospital-based cross-sectional study and according to the data of the hospital where the research was carried out, the total number of pregnancies in 2019 was 6128. Of these, 2267 were primigravida. With the assumption that the number of primigravida would stay the same in 2020, the study population was determined as 2267. The sample size was computed using the formula:


n=Nt2pq/d2(N-1)+t2pq

where ‘N’ is the population size; ‘n’ is the sample size; ‘p’ is the incidence (probability) of the event under investigation; ‘q’ is the incidence (probability) of the absence of the event under investigation; ‘t’ is the theoretical value found in the t table at a given degree of freedom; and the error level ‘d’ is the desired standard deviation (SD) according to the incidence of the event. Accordingly, with p=0.50, q=0.50, t=1.96, and d=0.05, the sample size was determined as 328 primigravida women.

The inclusion criteria for the study included: I) being over the age of 18 years; II) confirm to participate in the study; and III) being primigravida. The exclusion criteria for the study included: I) being under the age of 18 years; II) refusal to participate in the study; III) being pregnant before; and V) willingness to leave study.

The data for the study were collected after local ethics committee approval, written consent from the hospital administration, verbal and written consent from the patients were obtained. The protocol was approved by The Kafkas University Faculty of Medicine Ethics Committee (No: 80576354-050-99/251, date: 25.11.2020). The study was carried out in accordance with the principles of the Declaration of Helsinki.

The data collection form was prepared by the researchers based on literature. The form consists of 2 parts; the first part focused on sociodemographic characteristics, and the second part consisted of the physical examination findings by the physician. The dependent variable of the study is anorectal diseases in primigravida. The independent variable of the study is sociodemographic characteristics and physical examination findings of the mother and neonate.

The study data were collected from pregnant women who applied to Gynecology and Obstetrics Polyclinic, Kars Harakani State Hospital, and General Surgery Polyclinic, Kafkas University, Kars, Turkey, between November 2020 and August 2021. The data was collected by an obstetrician and general surgeon who carried out the study. The pregnant women included in the study were examined and surveyed 4 times: in the second and third trimesters, on the day after delivery, and in the first month after delivery.

Pregnant women included in the study and who were found to have perianal diseases (hemorrhoids and anal fissures) received sitting bath and a combination of tribenoside and lidocaine treatment (RECORDATI, Turkey).

### Statistical analysis

The Statistical Package for the Social Sciences, version 21.0 (IBMCorp, Armonk, NY, USA) analysis program was used for data analysis. Chi-square test was used in the analysis of the data determined by counting. Variables which were statistically significant (*p*<0.05) in the Chi-square test were included in the backward logistic regression analysis.

## Results

**Figure 1:**
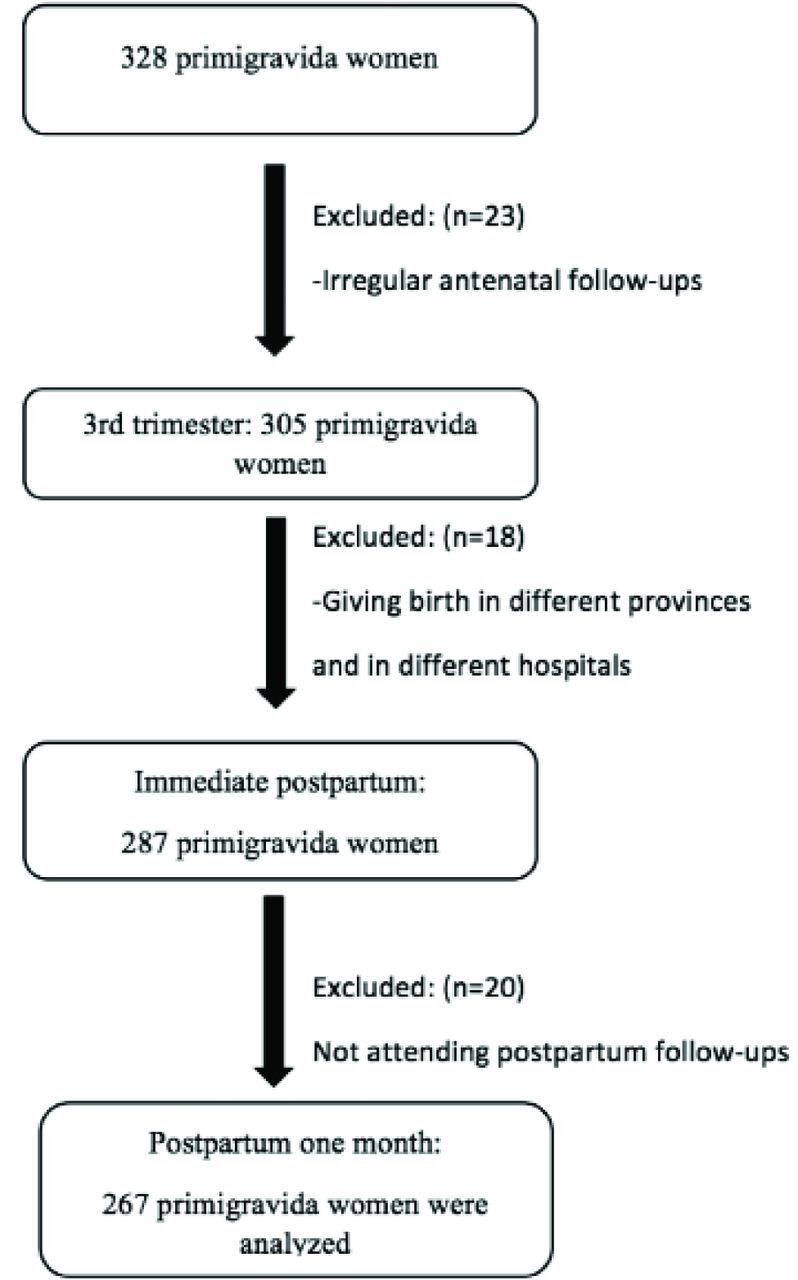
- Flowchart of the study population.

Of the 328 primigravida women included in the study, 23 (7%) missed their follow-up in the third trimester, 18 (5.5%) missed their follow-up on the day after the delivery, and 20 (6.1%) missed their follow-up in the first month after delivery. Thus, the study included 267 primigravida women. Of these, 38.6% (n=103) had perianal diseases and 61.4% (n=164) did not have perianal diseases (Figure 1 & Table 1). The reasons for 61 patients’ exclusion from the study in total were irregular antenatal follow-ups, giving birth in different provinces and in different hospitals, and not attending postpartum follow-ups.

As shown in Table 1, bleeding was most commonly observed in the 3^rd^ trimester and at the time of delivery (22.3%), whereas itching was most commonly observed in the 3^rd^ trimester (25.2%), and anal pain (44.7%), anal swelling (35%), and constipation (41.7%) were most common in the 3^rd^ trimester.

In Tables 2 & 3, a total of 267 patients in the final state of our study were divided into those with and without perianal disease, and no trimester grouping was made. Sociodemographic and various characteristics and maternal and neonatal characteristics were compared as risk factors in groups with and without perianal disease, which is our dependent variable.

There was no significant difference in the incidence of perianal diseases with respect to age (*p*=0.098), place of residence (*p*=0.228), educational status (*p*=0.105), health insurance (*p*=0.134), employment status (*p*=0.229), smoking status (*p*=0.304), body mass index (*p*=0.241), participating in sports (*p*=0.907), delivery method (*p*=0.654), episiotomy (*p*=0.218), and baby’s head circumference (*p*=0.175). However, a significant difference was found in the incidence of perianal diseases in terms of family income (*p*=0.008), perianal disease history (*p*=0.001), fiber consumption (*p*=0.002), constipation (*p*=0.001), birth weight (*p*=0.001), and duration of the pushing stage (*p*=0.001; Tables 2 & 3).

Women with at least one of the symptoms such as bleeding, itching, anal pain, anal swelling, and constipation were considered to have perianal disease. The number of primigravids with perianal disease was determined as 103.

Variables that were found to be statistically significant among the independent variables shown in Tables 2 & 3 were included in the logistic regression analysis. Logistic regression analysis results are shown in Table 4. In the logistic regression analysis of the independent variables which were significant in the pairwise analyses, only the duration of the pushing stage was found to be a risk factor during delivery. When the independent variables in both Tables 2 & 3 were evaluated together, family income and birth weight were no longer identified as risk factors. As a result, when the independent variables that were statistically significant in pairwise comparisons were considered in the logistic regression analysis, the incidence of perianal disease was found to be 4.917 times (confidence interval [CI]: [2.134-11.327]) higher in those with perianal disease compared with those without, 2.936 times (CI: [1.584-5.439]) higher in those who did not consume fiber-rich foods compared with those who did, 9.512 times (CI: [4.583-19.742]) higher in those with constipation compared with those without, and 23.721 times (CI: [5.363-104.915]) higher in those whose pushing stage duration was above average compared with those whose pushing stage duration was below average (Table 4).

In Model 1, income, history, consumption of fibrous foods, and constipation, which resulted in statistically significant results, were included in the logistic regression analysis. All independent variables were determined as risk factors. In Model 2, duration of the pushing stage and birth weight were statistically significant. Two independent variables were included in the logistic regression analysis. Only the duration of the pushing stage was significant. In Model 3, Model 1 and Model 2 were simultaneously included in the logistic regression analysis. Here, the income effect in Model 1 was no longer a risk factor (Table 4).

**Table 1:** - Distribution of perianal disease symptoms over time periods and based on the level of participation in the study (N=103).

Symptoms	2^nd^ trimester	3^rd^ trimester	Immediate postpartum	One month postpartum
Bleeding	21 (20.4)	23 (22.3)	23 (22.3)	17 (16.5)
Itching	24 (23.3)	26 (25.2)	21 (20.4)	13 (12.6)
Anal pain	24 (23.3)	29 (28.2)	46 (44.7)	21 (20.4)
Anal swelling	12 (11.7)	27 (26.2)	37 (35.9)	17 (16.5)
Constipation	29 (28.2)	37 (35.9)	43 (41.7)	19 (18.4)

Values are presented as a number and precentage (%). Percentages are based on multiple responses.

**Table 2:** - Distribution of sociodemographic and other characteristics with respect to perianal diseases.

Variables	Perianal disease*		Total^†^	X^2^	*P*-values
	Yes	No			
***Age***					
≥19	24 (49.0)	25 (51.0)	49 (18.4)	2.741	0.098
≤20	79 (36.2)	139 (63.8)	218 (81.6)		
***Place of residence***					
Urban areas	73 (36.5)	127 (63.5)	200 (74.9)	1.451	0.228
Rural areas	30 (44.8)	37 (55.2)	67 (25.1)		
***Education***					
≤8	40 (45.5)	48 (54.5)	88 (33.0)	2.620	0.105
≥9	63 (35.2)	116 (64.8)	179 (67.0)		
***Health insurance***					
No	9 (56.2)	7 (43.8)	16 (6.0)	2.244	0.134
Yes	94 (37.5)	157 (62.5)	251 (94.0)		
***Household income***					
Sufficient	16 (24.6)	49 (75.4)	65 (24.3)	7.068	0.008
Insufficient	87 (43.1)	115 (56.9)	202 (75.7)		
***Working status***					
Unemployed	78 (40.8)	113 (59.2)	191 (71.5)	1.448	0.229
Employed	25 (32.9)	51 (67.1)	76 (28.5)		
***Smoking***					
Smoker	15 (46.9)	17 (53.1)	32 (12.0)	1.057	0.304
Non-smoker	88 (37.4)	147 (62.6)	235 (88.0)		
***BMI (kg/m*** ^2^ ***)***					
≤25	24 (32.9)	49 (67.1)	73 (27.3)	1.378	0.241
>25	79 (40.7)	115 (59.3)	194 (72.7)		
***Sports and exercise***					
Yes	6 (40.0)	9 (60.0)	15 (5.6)	0.014	0.907
No	97 (38.5)	155 (61.5)	252 (94.4)		
***History of perianal disease***					
Yes	32 (65.3)	17 (34.7)	49 (18.4)	18.095	0.001
No	71 (32.6)	147 (67.4)	218 (81.6)		
***Consumption of fibrous foods***					
Occasional	61 (48.4)	65 (51.6)	126 (47.2)	9.742	0.002
Daily	42 (29.8)	99 (70.2)	141 (52.8)		
***Constipation***					
No	15 (14.0)	92 (86.0)	107 (40.1)	45.447	0.001
Yes	88 (55.0)	72 (45.0)	160 (59.9)		
Total	103 (38.6)	164 (61.4)	267 (100.0)		

Values are presented as a number and precentage (%). ^*^Row percentage, ^†^column percentage, BMI: body mass index

**Table 3:** - Distribution of maternal and infant characteristics with respect to perianal diseases.

Variables	Perianal disease*		Total^†^	X^2^	*P*-values
	Yes	No			
***Birth method***					
Cesarean	50 (40.0)	75 (60.0)	125 (46.8)	0.201	0.654
Vaginal	53 (37.3)	89 (62.7)	142 (53.2)		
***Episiotomy***					
No	77 (36.7)	133 (63.3)	210 (78.7)	1.515	0.218
Yes	26 (45.6)	31 (54.4)	57 (21.3)		
***Birth weight (grams)***					
>3186	22 (75.9)	7 (24.1)	29 (10.9)	19.087	0.001
≤3186	81 (34.0)	157 (66.0)	238 (89.1)		
***Head circumference (centimeter)***					
>33.2	18 (48.6)	19 (51.4)	37 (13.9)	1.839	0.175
≤33.2	85 (37.0)	145 (63.0)	230 (86.1)		
***Duration of the pushing stage (minutes)***					
>18	19 (86.4)	3 (13.6)	22 (8.2)	23.106	0.001
≤18	84 (34.3)	161 (65.7)	245 (91.8)		
Total	103 (38.6)	164 (61.4)	267 (100.0)		


Values are presented as a number and precentage (%). ^*^Row percentage, ^†^column percentage

**Table 4:** - Logistic regression analysis results.

Variables	Perianal disease					
	2^nd^ and 3^rd^ trimester (Model 1)		Immediate postpartum (Model 2)		one month postpartum (Model 3)	
	*P*-values	OR (95% CI)	*P*-values	OR (95% CI)	*P*-values	OR (95% CI)
***Income***						
Insufficient	0.014	1 (0.207-0.838)			0.113	0.559 (0.272-1.148)
Sufficient		1 (ref)				1 (ref)
***History***						
Yes	0.001	5.955 (2.569-13.804)			0.001	4.917 (2.134-11.327)
No		1 (ref)				1 (ref)
***Consumption of fibrous foods***						
No consumption	0.001	2.757 (1.520-4.999)			0.001	2.936 (1.584-5.439)
Consuming		1 (ref)				1 (ref)
***Constipation***						
Yes	0.001	7.407 (3.822-14.353)			0.001	9.512 (4.583-19.742)
No		1 (ref)				1 (ref)
***Duration of the pushing stage***						
Above average			0.001	12.139 (3.492-42.195)	0.001	23.721 (5.363-104.915)
Average and below				1 (ref)		1 (ref)
***Birth weight***						
Above average			0.630	1.454 (0.318-6.652)	0.827	0.818 (0.135-4.953)
Average and below				1 (ref)		1 (ref)

OR: odds ratio, CI: confidence interval, ref: reference

## Discussion

Many etiological factors, such as hard stools, chronic constipation, prolonged straining, increased vascular blood flow due to increased intra-abdominal pressure, absence of valves in veins and drainage vessels in hemorrhoids, lack of continuous pelvic floor support, genetic factors, damage to the internal anal sphincter, and pregnancy are responsible for the development of hemorrhoids.^3,8^ Moreover, a study on 61 primigravid women showed that pregnancy had a direct effect on the development of perianal diseases.^9^

Additionally, a study on 280 pregnant women reported that perianal diseases were observed in 43.9% of the women.^10^ In a study carried out on 217 pregnant women in England, the distribution of the incidence of perianal diseases according to trimesters varied between 16-43%.^11^ In the present study, the incidence of perianal diseases in primigravid women was determined as 38.6%.

Having a history of perianal disease, no consumption of fibrous food, constipation, and a prolonged pushing stage were found to be important and independent risk factors for perianal diseases during pregnancy and the perinatal period. In the present study, the presence of perianal disease was 4.917 times higher in patients with a history of perianal disease compared with those without. In another study, 57.2% of 495 pregnant women were reported to have had a history of perianal disease before the current pregnancy.^8^ In a study carried out on 280 pregnant women in Lithuania, the history of perianal disease before pregnancy was found to be 1.377 times higher in those with perianal disease during pregnancy and in the perinatal period.^10^ Consistent with the present study results, a study carried out on 94 pregnant women in Belgium reported that a history of perianal disease before pregnancy was found to be an important risk factor both during pregnancy and in the period up to the 3^rd^ month after delivery.^12^ In a study carried out on patients who underwent surgery for hemorrhoids during pregnancy, a history of perianal disease before pregnancy was found to be a risk factor for perianal diseases.^13^

One of the factors affecting the development of perianal disease is diet.^3,14^ A study carried out in India, where fiber consumption is very high, showed that the incidence of hemorrhoids was only 1.8%.^15^ Contrary to a study’s reports that a fibrous diet is not more effective than placebo in terms of therapeutic success, multiple studies support the malnutrition theory, which states that consuming insufficient amounts of fibrous foods during pregnancy leads to the development of perianal diseases in pregnant women.^8,11,16,17^ In the present study, the incidence of perianal disease was 2.936 times higher in those who did not consume fibrous food compared with those who did.

Constipation is an important risk factor for the development of perianal diseases during pregnancy and in the postpartum period. The pathophysiology of constipation during pregnancy involves high progesterone levels causing decreased small bowel and colon motility, which can worsen with decreased water consumption as well as lack of a fiber-rich diet and the pressure of the uterus on the rectosigmoid colon in the later stages of pregnancy.^6,18-21^ There are limited prospective studies involving prepartum and postpartum processes similar to the present study. The studies of Ferdinande et al^12^ and Poskus et al^10^ are similar to the present study in terms of several aspects. These studies revealed that constipation is an independent risk factor in the development of perianal disease in pregnant women in all the stages. In the study carried out by Poskus et al,^10^ the incidence of perianal disease was 18.975 times higher in patients with constipation compared with those without. The present study found that the incidence of perianal disease was 9.512 times higher in those with constipation than those without in the prepartum and postpartum period.

Perianal diseases have been associated with difficult delivery, and studies indicate that a prolonged pushing time may be a risk factor for the development of perianal disease.^22-24^ There is a study stating that the average duration of the pushing stage is 13.4 minutes and that prolonged pushing over 20 minutes is a risk factor for the development of perianal disease.^10^ In the present study, the mean duration of the pushing stage was found to be 18 minutes, and the incidence of perianal disease was found to be 23.721 times higher in patients with an above average duration of the pushing stage compared with those with a below average duration of the pushing stage.

### Study strengths & limitations

Among the limitations, the absence of long-term follow-ups after the first month postoperatively, and the fact that the sudy did not cover the entire region. The strengths of the study were that the study population consisted of primigravid pregnant women; it included all pregnancy and postpartum first month follow-ups.

In conclusion, in primiparous pregnants; the risk of perianal disease increases in those who have perianal disease before pregnancy, those who do not consume fibrous food, those who have constipation, and those who have a long pushing period.

## References

[1] Hyams L , Philpot J. An epidemiological investigation of hemorrhoids. Am J Proctol 1970; 21: 177-193. 5425245

[2] Mirhaidari SJ , Porter JA , Slezak FA. Thrombosed external hemorrhoids in pregnancy: a retrospective review of outcomes. Int J Colorectal Dis 2016; 31: 1557-1559. 27029798 10.1007/s00384-016-2565-y

[3] Avsar AF , Keskin HL. Haemorrhoids during pregnancy. J Obstet Gynaecol 2010; 30: 231-237. 20373920 10.3109/01443610903439242

[4] Staroselsky A , Nava-Ocampo AA , Vohra S , Koren G. Hemorrhoids in pregnancy. Can Fam Physician 2008; 54: 189-190. 18272631 PMC2278306

[5] Ansara D , Cohen MM , Gallop R , Kung R , Schei B. Predictors of women’s physical health problems after childbirth. J Psychosom Obstet Gynaecol 2005; 26: 115-125. 16050537 10.1080/01443610400023064

[6] Shin GH , Toto EL , Schey R. Pregnancy and postpartum bowel changes: constipation and fecal incontinence. Am J Gastroenterol 2015; 110: 521-529. 25803402 10.1038/ajg.2015.76

[7] Vazquez JC. Constipation , haemorrhoids, and heartburn in pregnancy. BMJ Clin Evid 2010; 2010: 1411. PMC3217736 21418682

[8] Shirah BH , Shirah HA , Fallata AH , Alobidy SN , Hawsawi MMA. Hemorrhoids during pregnancy: Sitz bath vs. ano-rectal cream: a comparative prospective study of 2 conservative treatment protocols. Women Birth 2018; 31: e272-e277. 29055673 10.1016/j.wombi.2017.10.003

[9] Beksac K , Aydin E , Uzelpasaci E , Akbayrak T , Ozyuncu O. Hemorrhoids and related complications in primigravid pregnancy. JCOL 2018; 38: 179-182.

[10] Poskus T , Buzinskienė D , Drasutiene G , Samalavicius NE , Barkus A , Barisauskiene A , et al. Haemorrhoids and anal fissures during pregnancy and after childbirth: a prospective cohort study. BJOG 2014; 121: 1666-1671. 24810254 10.1111/1471-0528.12838

[11] Ferdinande K , Dorreman Y , Roelens K , Ceelen W , De Looze D. Anorectal symptoms during pregnancy and postpartum: a prospective cohort study. Colorectal Dis 2018; 20: 1109-1116. 29972721 10.1111/codi.14324

[12] Yabanoglu H. [Is surgical treatment of complicated hemorrhoids during pregnancy safe? A retrospective analysis of 13 patients and literature review]. J of Cukurova Anesthesia and Surg Sci 2019; 2: 26-32. [In Turkish]

[13] Odabas K , Taspinar A. [Constipation in pregnancy and its relationship with quality of life]. Nurs Health Sci 2020; 23: 250-258. [In Turkish]

[14] Bhatia JC , Cleland J. Self-reported symptoms of gynecological morbidity and their treatment in south India. Stud Fam Plann 1995; 26: 203-216. 7482678

[15] Yang J , Wang HP , Zhou L , Xu CF. Effect of dietary fiber on constipation: a meta analysis. World J Gastroenterol 2012; 18: 7378-7383. 23326148 10.3748/wjg.v18.i48.7378 PMC3544045

[16] Nisar PJ , Scholefield JH. Managing haemorrhoids. BMJ 2003; 327: 847-851. 14551102 10.1136/bmj.327.7419.847 PMC214027

[17] Unadkat SN , Leff DR , Teoh TG , Rai R , Darzi AW , Ziprin P. Anorectal symptoms during pregnancy: how important is trimester? Int J Colorectal Dis 2010; 25: 375-379. 19921218 10.1007/s00384-009-0845-5

[18] Davis BR , Lee-Kong SA , Migaly J , Feingold DL , Steele SR. The American Society of Colon and Rectal Surgeons Clinical Practice Guidelines for the Management of Hemorrhoids. Dis Colon Rectum 2018; 61: 284-292. 29420423 10.1097/DCR.0000000000001030

[19] Meinds RJ , Van Meegdenburg MM , Trzpis M , Broens PM. On the prevalence of constipation and fecal incontinence, and their co-occurrence, in the Netherlands. Int J Colorectal Dis 2017; 32: 475-483. 27913883 10.1007/s00384-016-2722-3 PMC5355501

[20] Bimba K , Patil GL , Shridevi AS , Praveena SN , Asha B , Mandava S , et al. Prevalence of constipation in pregnancy- a prospective study at a tertiary care hospital. Journal of Gynecology 2017; 1: 1-11.

[21] Cauley CE , Savitt LR , Weinstein M , Wakamatsu MM , Kunitake H , Ricciardi R , et al. A quality-of-life comparison of 2 fecal incontinence phenotypes: isolated fecal incontinence versus concurrent fecal incontinence with constipation. Dis Colon Rectum 2019; 62: 63-70. 30451749 10.1097/DCR.0000000000001242

[22] Thompson JF , Roberts CL , Currie M , Ellwood DA. Prevalence and persistence of health problems after childbirth: associations with parity and method of birth. Birth 2002; 29: 83-94. 12051189 10.1046/j.1523-536x.2002.00167.x

[23] Gjerdingen DK , Froberg DG , Chaloner KM , McGovern PM. Changes in women’s physical health during the first postpartum year. Arch Fam Med 1993; 2: 277-283. 8252148 10.1001/archfami.2.3.277

[24] Abramowitz L , Sobhani I , Benifla JL , Vuagnat A , Daraï E , Mignon M , et al. Anal fissure and thrombosed external hemorrhoids before and after delivery. Dis Colon Rectum 2002; 45: 650-655. 12004215 10.1007/s10350-004-6262-5

